# Social Isolation Is Associated With Rapid Kidney Function Decline and the Development of Chronic Kidney Diseases in Middle-Aged and Elderly Adults: Findings From the China Health and Retirement Longitudinal Study (CHARLS)

**DOI:** 10.3389/fmed.2021.782624

**Published:** 2021-12-02

**Authors:** Weiran Zhou, Yang Li, Yichun Ning, Shaomin Gong, Nana Song, Bowen Zhu, Jialin Wang, Shuan Zhao, Yiqin Shi, Xiaoqiang Ding

**Affiliations:** ^1^Division of Nephrology, Zhongshan Hospital, Fudan University, Shanghai, China; ^2^Shanghai Medical Center of Kidney Disease, Shanghai, China; ^3^Shanghai Institute of Kidney and Dialysis, Shanghai, China; ^4^Shanghai Key Laboratory of Kidney and Blood Purification, Shanghai, China; ^5^Hemodialysis Quality Control Center of Shanghai, Shanghai, China

**Keywords:** social isolation, chronic kidney disease, glomerular filtration rate, CHARLS, Chinese middle-aged and older adults

## Abstract

**Background:** There is limited evidence on the relationship between social isolation and renal outcomes. To address this gap, this study estimated the prospective relationship of social isolation with rapid kidney function decline and the development of chronic kidney disease (CKD) in middle-aged and elderly Chinese with normal kidney function.

**Methods:** We analyzed data from 3,031 participants aged ≥ 45 years with baseline estimated glomerular filtration rates (eGFR) ≥ 60 ml/min/1.73 m^2^. All data were obtained from the 2011 and 2015 waves of the Chinese Longitudinal Study of Health and Retirement (CHARLS). eGFR was estimated based on a combination of serum creatinine and cystatin C. The primary outcome was rapid decline in renal function, as defined by an eGFR decrease of **>** 5 ml/min/1.73 m^2^ per year, while the secondary outcome was the development of CKD, as defined by an eGFR decrease to a level < 60 ml/min/1.73 m^2^.

**Results:** During the follow-up of 4 years, 258 (8.5%) participants experienced a rapid decline in renal function, while 87 (2.9%) developed CKD. In the fully adjusted model, high social isolation was significantly related to an increased risk of experiencing a rapid decline in renal function (OR 1.805, 95% CI 1.310–2.487) and CKD onset (OR 1.842, 95% CI 1.084–3.129). Among the five components of social isolation, being unmarried, not participating in social activities, and living alone independently predicted declined renal function.

**Conclusions:** Social isolation is significantly associated with the risk of rapid eGFR decline and CKD onset in middle-aged and older adults with normal kidney function in China.

## Introduction

The social networks are composed of various interactive relationships between individuals and organizations. These relationships facilitate the establishment of self-cognition and enable social support. However, older adults may gradually become separated from their social network due to factors such as retirement, disease, reduced family size, and many others. More specifically, social isolation is defined as the lack of social relationships and low levels of contact with family, friends, the community, and general social environment (1). As the national population continues to age, social isolation is becoming an increasingly serious concern in China.

There is extensive evidence that social isolation leads to both a lower quality-of-life and higher incidence of several psychological and physical diseases (2, 3). Notably, the risk of death from social isolation is comparable to that imposed by some well-established clinical factors, including alcoholism, smoking, and obesity (4). Social isolation also predicts a greater risk of cognitive decline (5), cardiovascular disease (6–8), cerebrovascular disease (7, 8), and all-cause mortality (9, 10). As for health behaviors and self-care, social isolation is associated with increased tobacco use, less vegetable and fruit intake, and less physical activity (11).

The burdens and costs resulting from chronic kidney disease (CKD) have recently begun to increase across the globe (12). This makes it imperative to identify the modifiable risk factors for CKD, especially those that may quickly be addressed to prevent or slow the progression of declining kidney function. Apart from the known clinical factors, new evidence continues to show that social factors may also affect the onset and progression of CKD. For example, Dunkler et al. suggested that a greater number of friends reduced the incidence of and delayed the progression of CKD in individuals with type 2 diabetes (13). Tomaka et al. found that family support independently predicted the outcomes of kidney disease in Hispanic participants (14). Zhang et al. emphasized the associations between depressive symptoms and the risk of kidney function decline; of particular note, loneliness was found to be an independent risk factor (15). In sum, the literature shows that increased attention should be paid to the impacts of social networks on CKD.

To date, there have been no nationally representative studies aimed at assessing the influence of social isolation on the progression of CKD. To address this gap, this prospective study investigated the relationships between social isolation, rapid kidney function decline, and CKD onset in a sample of middle-aged and older adults in China based on four-year follow-up data from the China Health and Retirement Longitudinal Study (CHARLS).

## Methods

### Study Design and Participants

**Figure 1:**
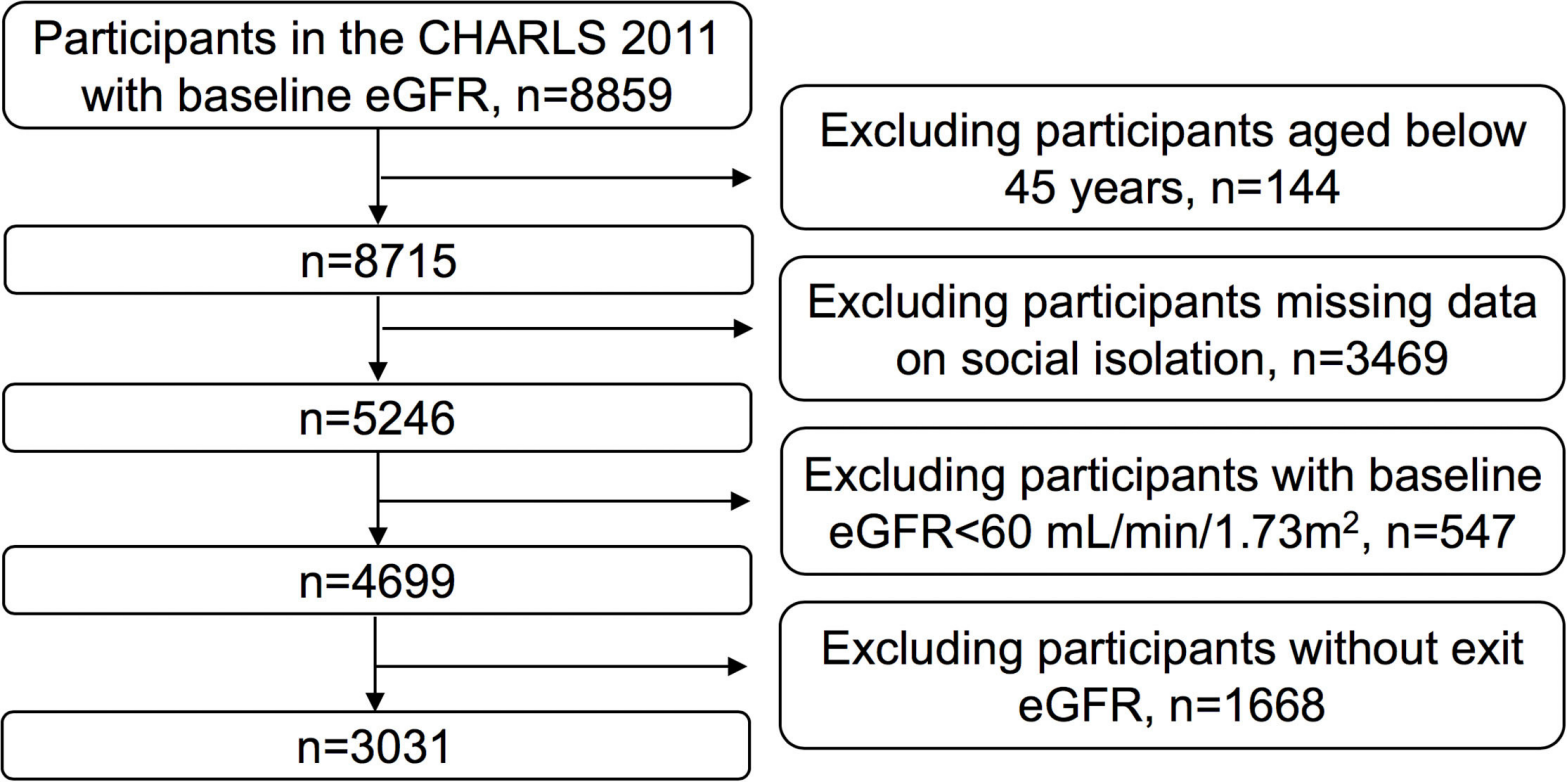
Flow diagram of participants for the study.

CHARLS is a nationally representative longitudinal survey that covers 150 counties and 450 communities across 28 provinces in mainland China. The data resource profile and detailed information about blood sample taken has been reported previously(16–18). The survey aims to establish a high quality set of micro-databases that represent middle-aged and elderly individuals in China and their families. The baseline survey was conducted in 2011, with three follow-up surveys conducted in 2013, 2015, and 2018. Ethical approval was received at Peking University (IRB00001052–11015). This study used data from the 2011 and 2015 waves of CHARLS, including information from 8,859 participants who provided baseline blood data related to kidney function. Based on our study criteria, we excluded 5,828 of these participants, including 144 who were aged <45 years, 3,469 who did not provide data on social isolation, 547 with a baseline estimated glomerular filtration rate (eGFR) below 60 mL/min/1.73 m^2^, and 1,668 without exit eGFR. As such, a total of 3,031 participants were ultimately included for analysis (Figure 1).

### Assessment of Social Isolation

The social isolation index was based on the five following items: being unmarried (never married, divorced, and widowed), living alone, having in-person or phone/email contact with their children less often than once per week, living in a rural area, and no participation in social activities over the previous month (e.g., visiting social clubs, interacting with friends, voluntary work, playing cards). Total scores ranged from 0 to 5, with higher scores indicating greater social isolation. We then categorized participants based on a cutoff score of 2, thereby resulting in groups for low (<2) and high (≥2) social isolation (10, 19).

### Assessment of Kidney Function

The Chronic Kidney Disease Epidemiology Collaboration (CKD-EPI) creatinine-cystatin C equation (20) was used to calculate eGFR, as follows:

eGFR (mL/min/1.73m^2^) = 135 × min(Cr/k, 1)^α^ × max(Cr/k, 1)^−0.601^ × min(Cys/0.8, 1)^−0.375^ × max(Cys/0.8, 1)^−0.711^ ×0.995^age^ ×0.969[if female]

where Cr refers to serum creatinine measured in mg/dL and CysC refers to serum cystatin C measured in mg/liter. k is 0.7 for females and 0.9 for males. α is −0.248 for females and −0.207 for males.

### Outcomes

The primary outcome was rapid eGFR decline, as defined by a decline of > 5 mL/min/1.73 m^2^/year; this cutoff has been used in previous studies (15, 21). The secondary outcome was the development of CKD, as defined by an eGFR decrease to a level < 60 ml/min/1.73 m^2^.

### Assessment of Covariates

Participants also provided their demographic data and health-related behavioral information (e.g., tobacco use, alcohol consumption, and medication usage) via the CHARLS survey. Each participant had their blood pressure measured three times using a HEM-7200 electronic monitor (Omron, Dalian); these measurements were then averaged and recorded. In this context, hypertension was defined based on systolic blood pressure ≥ 130 mmHg and/or diastolic blood pressure ≥ 80 mmHg or the use of antihypertensive drugs. Next, diabetes was described as fasting glucose ≥ 126 mg/dl, random glucose ≥ 200 mg/dl, hemoglobin A1c ≥ 6.5%, or the use of hypoglycemic drugs. The 10-question version of the Center for Epidemiological Research Depression Scale (CES-D) was used to assess depressive symptoms, which were considered present based on CES-D scores ≥ 10 (22). Drinking was defined as drinking more than once a month currently or in the past. Smoking was defined as having history of smoking including former smoking and current smoking. More specifically, having a response of “Drink more than once a month” to the question “Did you drink any alcoholic beverages, such as beer, wine, or liquor in the past year? How often?” or having a response of “I used to drink more than once a month” to the question “Did you ever drink alcoholic beverages in the past? How often?” were classified into drinking. Having a “yes” response to the question “Have you ever chewed tobacco, smoked a pipe, smoked self-rolled cigarettes, or smoked cigarettes/cigars?” was classified into smoking. The questionnaires can be found at the CHARLS website (http://charls.pku.edu.cn/pages/data/2011-charls-wave1/zh-cn.html).

### Statistical Analyses

The data were expressed as means ± standard deviations (SDs) for continuous variables and as numbers and percentages for categorical variables. The level of social isolation was analyzed as a dichotomous variable. The Student's t-test or Pearson χ^2^ test were used to compare participant characteristics based on low or high levels of social isolation. The univariate and multivariate logistic regression models were used to describe the relationships between social isolation and kidney outcomes. For the multivariate analysis, adjusted covariates included age, sex, body mass index, smoking status, systolic blood pressure, diastolic blood pressure, glucose, total cholesterol, triglycerides, HDL cholesterol, eGFR, uric acid, and CES-D scores. All statistical analyses were conducted using IBM SPSS version 23.0 (IBM Corporation, Armonk, NY, USA).

## Results

### Characteristics of Participants

Table 1 shows the participant baseline characteristics based on their social isolation category (low or high). Among the 3,031 total analyzed participants, 1,353 were placed into the low social isolation group, while 1,678 were placed into the high social isolation group. The following baseline values were obtained: average age of 59.8 ± 9.2 years, 51.2% female, and mean eGFR of 87.6 ± 14.6 ml/min/1.73 m^2^. Participants in the high social isolation group were generally older and less educated than those in the low social isolation group; they also tended to have higher CES-D scores, lower body mass index values, and lower baseline eGFR. Supplementary Table 1 shows baseline characteristics for the participants we excluded from analysis. Compared with those who were excluded from the original sample (*n* = 5,828), participants included in the final analysis were younger, had higher CES-D scores, and were more educated.

**Table 1:** Baseline characteristics of participants (*n* = 3,031) according to social isolation categories.

	**Total**	**Low social isolation**	**High social isolation**	***p*** **-value**
Unweighted N	3,031	1,353	1,678	
Age (year)	59.8 ± 9.2	57.6 ± 8.5	61.5 ± 9.3	**<0.001**
Sex (female), N (%)	1,553 (51.2%)	683 (50.5%)	870 (51.8%)	0.454
Elementary school education or above (%)	1,628 (53.7%)	889 (65.7%)	739 (44.0%)	**<0.001**
Hypertension (%)	1,393 (46.0%)	589 (43.5%)	804 (47.9%)	**0.016**
Diabetes (%)	403 (13.3%)	191 (14.1%)	212 (12.6%)	0.232
Body mass index (kg/m^2^)	23.4 ± 3.6	24.2 ± 3.7	22.9 ± 3.5	**<0.001**
Systolic blood pressure (mmHg)	130.5 ± 21.6	129.1 ± 21.0	131.5 ± 22.0	**0.005**
Diastolic blood pressure (mmHg)	75.4 ± 12.0	75.7 ± 11.9	75.2 ± 12.1	0.359
eGFR (mL/min/1.73 m^2^)	87.6 ± 14.6	89.4 ± 15.1	86.0 ± 14.0	**0.001**
Uric acid (mg/dL)	4.4 ± 1.2	4.5 ± 1.2	4.3 ± 1.2	**<0.001**
Glucose (mg/dL)	109.2 ± 32.4	110.1 ± 33.6	108.6 ± 31.5	0.256
Glycated hemoglobin (%)	5.3 ± 0.8	5.3 ± 0.8	5.3 ± 0.8	0.204
Total cholesterol (mg/dL)	194.2 ± 37.5	194.9 ± 37.4	193.7 ± 37.6	0.836
HDL cholesterol (mg/dL)	51.3 ± 15.3	49.7 ± 14.9	52.6 ± 15.5	0.067
LDL cholesterol (mg/dL)	117.3 ± 34.6	117.7 ± 35.3	117.0 ± 34.1	0.382
Triglycerides (mg/dL)	132.1 ± 97.5	141.6 ± 111.5	124.4 ± 83.9	**<0.001**
Drinking (%)	782 (25.8%)	347 (25.6%)	435 (26.0%)	0.862
Smoking (%)	1,239 (40.9%)	532 (39.3%)	707 (42.1%)	0.117
Depressive symptoms score	9.8 ± 5.5	9.4 ± 5.0	10.2 ± 5.8	**<0.001**
**Baseline social isolation**				
Not married (%)	590 (19.5%)	2 (0.1%)	588 (35.1%)	**<0.001**
Less than weekly contact with children (%)	305 (10.1%)	9 (0.7%)	296 (17.6%)	**<0.001**
Live in the rural area (%)	2,482 (84.1%)	693 (66.6%)	1,431 (96.3%)	**<0.001**
Not participate in social activities (%)	1,440 (47.5%)	165 (12.2%)	1,275 (76.0%)	**<0.001**
Live alone (%)	584 (19.3%)	1 (0.0%)	445 (34.7%)	**<0.001**

*Data are shown as means ± standard deviation or numbers (percentages).*

^*^p Values correspond two-tailed t-test for continuous variables and chi-squared test for categorical variables.

*HDL Cholesterol, high density lipoprotein cholesterol; LDL Cholesterol, low density lipoprotein cholesterol; eGFR, estimated glomerular filtration rate*.

### Analysis of the Associations of Social Isolation With Primary and Secondary Outcomes

Based on the follow-up data, 258 (8.5%) participants underwent rapid declines in kidney function, with 87 (2.9%) having progressed to CKD. Table 2 shows the relationships between social isolation and CKD outcomes. In the fully adjusted model (Model 2), participants in the high social isolation group had higher overall risks for both rapid eGFR decline (odds ratio [OR] 1.805, 95% confidence interval[CI] 1.310–2.487) and CKD development (OR 1.842, 95% CI 1.084–3.129) than those in the low social isolation group. Table 3 shows the relationships between the five components of social isolation and rapid eGFR decline. Among these components, being unmarried (OR 1.760, 95% CI 1.199–2.583), not participating in social activities (OR 1.353, 95% CI 1.002–1.828), and living alone independently (OR 1.715, 95% CI 1.166–2.523) predicted rapid eGFR decline in the fully adjusted model. However, none of the five components independently predicted CKD development (Supplementary Table 2).

**Table 2:** The relationships of social isolation and CKD outcomes.

**CKD outcomes**	**Social isolation** **categories**	**Events/N (%)**	**Model 1**		**Model 2**	
			**OR** **(95% CI)**	***P*** **value**	**OR** **(95% CI)**	***P*** **value**
Rapid decline in kidney function	Low	103/1,353 (7.6%)	(Reference)		(Reference)	
	High	155/1,678 (9.2%)	1.787 (1.344–2.375)	**<0.001**	1.805 (1.310–2.487)	**<0.001**
Progression to CKD	Low	24/1,353 (1.8%)	(Reference)		(Reference)	
	High	63/1,678 (3.8%)	2.046 (1.269–3.300)	**0.003**	1.842 (1.084–3.129)	**0.024**

*Model 1 was adjusted for eGFR at baseline. Model 2 was adjusted for age, sex, body mass index, smoking status, drinking status, systolic BP, diastolic BP, glucose, total cholesterol, triglycerides, HDL cholesterol, eGFR, uric acid, and the depressive symptoms score*.

**Table 3:** The relationships of social isolation and its components with rapid eGFR decline.

	**Events/N (%)**	**Model 1**		**Model 2**	
		**OR** **(95% CI)**	***P*** **value**	**OR** **(95% CI)**	***P*** **value**
**Social isolation components**					
Not married					
No	204/2,441 (8.4%)	(Reference)		(Reference)	
Yes	54/590 (9.2%)	1.956 (1.385–2.764)	**<0.001**	1.760 (1.199–2.583)	**0.004**
**Less than weekly contact with children**					
No	231/2,726 (8.5%)	(Reference)		(Reference)	
Yes	27/305 (8.9%)	1.158 (0.743–1.805)	0.518	1.237 (0.776–1.973)	0.371
**Living in the rural area**					
No	43/549 (7.8%)	(Reference)		(Reference)	
Yes	215/2,482 (8.6%)	1.268 (0.874–1.841)	0.211	1.416 (0.916–2.189)	0.118
**Not participating in social activities**					
No	127/1,591 (8.0%)	(Reference)		(Reference)	
Yes	131/1,440 (9.1%)	1.373 (1.043–1.807)	**0.024**	1.353 (1.002–1.828)	**0.049**
**Living alone**					
No	205/2,447 (8.4%)	(Reference)		(Reference)	
Yes	53/584 (9.1%)	1.915 (1.353–2.710)	**<0.001**	1.715 (1.166–2.523)	**0.006**

*Model 1 was adjusted for eGFR at baseline. Model 2 was adjusted for age, sex, body mass index, smoking status, drinking status, systolic BP, diastolic BP, glucose, total cholesterol, triglycerides, HDL cholesterol, eGFR, uric acid, and the depressive symptoms score*.

### Stratified Analyses

We conducted a stratified logistic regression analysis to determine whether any variables had interactions that changed the impacts of social isolation on rapid eGFR decline (Table 4). None of the variables (i.e., age, gender, body mass index, educational level, smoking, drinking, hypertension, diabetes, cholesterol, uric acid or depression symptoms) significantly modified the association between high social isolation and rapid eGFR decline (*P* > 0.05 for all interactions).

**Table 4:** Effect of social isolation on the risk of rapid eGFR decline by subgroups.

	**Events/N (%)**	**OR (95% CI)**	***P*** **value**	***P*** **for** **interaction**
**Age**				0.924
<65 years	209/2,209 (9.5%)	1.841 (1.298–2.611)	**0.001**	
≥65 years	49/822 (6.0%)	2.285 (1.015–5.143)	**0.046**	
**Sex**				0.816
Male	105/1,478 (7.1%)	1.847 (1.153–3.044)	**0.011**	
Female	153/1,553 (9.9%)	1.793 (1.157–2.777)	**0.009**	
**Body mass index**				0.876
<24 kg/m^2^	127/1,613 (7.9%)	1.762 (1.132–2.741)	**0.012**	
≥24 kg/m^2^	103/1,118 (9.2%)	2.000 (1.241–3.222)	**0.004**	
**Educational level**				0.845
< Primary school	119/1,403 (8.5%)	2.188 (1.311–3.652)	**0.003**	
≥Primary school	139/1,628 (8.5%)	1.621 (1.052–2.498)	**0.029**	
**Smoking**				0.792
No	180/1,792 (10.0%)	1.790 (1.205–2.659)	**0.004**	
Yes	78/1,239 (6.3%)	1.903 (1.081–3.351)	**0.026**	
**Drinking**				0.097
No	187/2,249 (9.1%)	1.609 (1.106–2.340)	**0.013**	
Yes	71/782(9.1%)	2.447 (1.268–4.721)	**0.008**	
**Hypertension**				0.988
No	120/1,345 (8.9%)	1.815 (1.168–2.819)	**0.008**	
Yes	112/1,393 (8.0%)	1.920 (1.186–3.107)	**0.008**	
**Diabetes**				0.526
No	219/2,628 (8.3%)	2.088 (1.460–2.987)	**<0.001**	
Yes	39/403 (9.7%)	0.837 (0.377–1.861)	0.663	
**Total cholesterol**				0.781
<200 mg/dL	130/1,790 (7.3%)	1.793 (1.156–2.782)	**0.009**	
≥200 mg/dL	128/1,241 (10.3%)	1.807 (1.126–2.900)	**0.014**	
**Uric acid**				0.695
<4.2 mg/dL	145/1,471 (9.9%)	1.894 (1.223–2.934)	**0.004**	
≥4.2 mg/dL	113/1,560 (7.2%)	1.711 (1.059–2.766)	**0.028**	
**Depression symptoms**				0.321
No	129/1,549 (8.3%)	1.691 (1.079–2.651)	**0.022**	
Yes	129/1,482 (8.7%)	1.943 (1.226–3.079)	**0.005**	

*The model was adjusted, if not stratified, for age, sex, body mass index, smoking status, systolic BP, diastolic BP, glucose, total cholesterol, triglycerides, HDL cholesterol, eGFR at baseline, uric acid, and the depressive symptoms score*.

## Discussion

Compared with the traditional clinical risk factors for CKD, social isolation has received relatively less attention in the public health context. However, this prospective analysis demonstrated that high social isolation was significantly associated with an increased risk of both rapid eGFR decline and CKD development in middle-aged and older persons in China. To the best of our knowledge, this was the first study to report that social isolation status could be used to predict CKD progression, thus contributing to the literature on modifiable renal risk factors.

CKD has been reported to be associated with social isolation in older adults. Participants with CKD Stages 3b-5 had less social participation (23). Reduced kidney function was also independently associated with hearing loss, which may cause social isolation (24). The previous studies both revealed that CKD was associated with social isolation. However, the key message of our longitudinal study was that social isolation predicted worsening kidney function and CKD onset, which was in the other direction and indicated the possible reciprocal relationship between social isolation and CKD. Future multi-center studies should be conducted to validate the findings of our current study and confirm the reciprocal relationship between social isolation and CKD.

In recent years, the national population in China has exhibited accelerated aging. This has created some serious concerns for elderly persons, who are at a higher risk of isolation due to factors such as physical frailty (25, 26), hearing loss (27, 28), the deaths of family members (29, 30), and less frequent contact with children (30). Even more problematically, social isolation that begins earlier in life often becomes more pronounced in later years due to age-related diseases and widowhood, further highlighting the need for interventions (30). However, there is still a general lack of public awareness about social isolation in China. In fact, most findings on the adverse effects of social isolation have been produced in Western countries, and may therefore not fully pertain to the Chinese context (5, 31). In this regard, perspectives on social isolation may markedly differ according to race, religion, community function, and other demographic factors. For example, the Berkman-Syme Social Networks Index has widely been used in previous studies, but only includes four main types of social contact (32), including church group membership, which is not widely relevant in China.

In this study, we measured five dimensions of social isolation that reflect Chinese culture according to the previous CHARLS analysis (19). It is worth mentioning that the three-item index of social isolation including being unmarried, having less contact with their children, and no participation in social activities as well as the four-item index of social isolation including the three-item index plus living in rural were also computed in previous studies using CHARLS data (5, 33). We adopted the five-item index of social isolation as it incorporated living alone, which was documented as the component of social isolation in quite a lot studies (32–35). We also dichotomized scores at ≥2 vs. <2 points to indicate high vs. low social isolation based on previous studies (19), which converted social isolation to a categorical variable and provided convenience for clinical risk classification.

Among the five components of social isolation, we found that being unmarried, living alone, and not participating in social activities were independent risk factors for renal function decline. Notably, a previous study in the United States showed that both being unmarried and infrequent participation in religious activities were predictors of mortality (36). Meanwhile, adults who live alone have been found to have a higher risk of ischemic heart disease mortality (37, 38) as well as a higher risk of falling and general functional decline (39, 40). These factors point to the need for increased social support and specialized public services for elderly persons who are unmarried, live alone, and/or lack social networks, particularly including social visitations and psychological care.

Several mechanisms may be accountable for the impact of social isolation on physical health outcomes. For one, individuals who integrate into society may have more tangible resources pertaining to health care knowledge, thus promoting their ability to engage in self-care (41, 42). Moreover, socially isolated individuals typically experience less social pressure to seek medical care and have poorer medication compliance (43). Apart from directly influencing health-related behaviors, social relationships also affect health-related physiology. For example, socially isolated individuals have higher expressions of genes related to pro-inflammatory cytokine signaling and enhanced immune function in inflammatory-related diseases (44, 45). This means that social isolation may actually worsen the pro-inflammatory state of CKD. In animal studies, social isolation activates the hypothalamus-pituitary-adrenal (HPA) axis and alters the gut microbiome (46, 47). Further, the feelings of being isolated from others is related to lower serum albumin levels (48). Taken together, these reports show that social relationships influence health through several psychological, behavioral, and biological mechanisms. However, continued research is need to clarify the underlying biological mechanisms by which social isolation affects kidney functions.

This study also had several limitations. First, all information on social isolation was self-reported. Here, recall bias may have resulted in underestimated social participation behaviors due to memory loss in some elderly persons. Second, we excluded 3,469 participants who did not have social isolation data. In this regard, nonparticipation by socially isolated individuals who were therefore not detected may have affected the final results. Third, while the level of social isolation is relatively stable, the analysis was solely conducted according to baseline measurements, even though life events may have occurred during the follow-up period. Finally, this was an observational study, meaning that further investigation is needed to establish causality.

## Conclusions

Based on data from a large-scale representative survey, this study found that social isolation was significantly associated with rapid declines in kidney function and CKD onset. These findings emphasize the need for interventions aimed at mitigating the effects of social isolation while also pointing out the potential clinical importance of social integration, especially for preventing CKD.

## Data Availability Statement

The original contributions presented in the study are included in the article/Supplementary Material, further inquiries can be directed to the corresponding author/s.

## Author Contributions

WZ, YL, and YN for analysis and interpretation of data, statistical analysis, and drafting of the manuscript. SG, NS, BZ, and JW for statistical analysis and technical support. SZ, YS, and XD for study concept and design, analysis and interpretation of data, drafting of the manuscript, obtained funding, and study supervision. All authors read and approved the final manuscript.

## Funding

This research was supported by the National Natural Science Foundation of China and the Science (81870476, 81800596, 82103911, and 81800592); Shanghai Municipal Education Commission (2017-01-07-00-07-E00009); Shanghai ‘Rising Stars of Medical Talent’ Youth Development Program (YS); Shanghai Key Laboratory of Kidney and Blood Purification (14DZ2260200 and 20DZ2271600); Shanghai Medical Centre of Kidney (2017ZZ01015).

## Conflict of Interest

The authors declare that the research was conducted in the absence of any commercial or financial relationships that could be construed as a potential conflict of interest.

## Publisher's Note

All claims expressed in this article are solely those of the authors and do not necessarily represent those of their affiliated organizations, or those of the publisher, the editors and the reviewers. Any product that may be evaluated in this article, or claim that may be made by its manufacturer, is not guaranteed or endorsed by the publisher.
