# Supplementary material for: Social Isolation Is Associated With Rapid Kidney Function Decline and the Development of Chronic Kidney Diseases in Middle-Aged and Elderly Adults: Findings From the China Health and Retirement Longitudinal Study (CHARLS)
Source: Front Med (Lausanne). 2021 Dec 2;8:782624. doi: 10.3389/fmed.2021.782624 (PMC8674531; doi:10.3389/fmed.2021.782624)
Supplement: Supplementary file 1 [file Data_Sheet_1.docx]

**Supplemental Table 1. Characteristics of the included and excluded participants.**

| **Characteristics** | **Included participates** | **Excluded participates** | **p-value** |
| --- | --- | --- | --- |
| Unweighted N | 3031 | 5828 |  |
| Age (year) | 59.8±9.2 | 60.7±10.8 | **<0.001** |
| Sex (female), N (%) | 1553(51.2%) | 3148(54.0%) | **0.013** |
| Body mass index (kg/m^2^) | 23.4±3.6 | 23.5±3.5 | 0.241 |
| Systolic blood pressure (mmHg) | 130.5±21.6 | 131.8±22.1 | **0.014** |
| Diastolic blood pressure (mmHg) | 75.4±12.0 | 75.7±12.2 | 0.388 |
| Drinking (%) | 782(25.8%) | 1402(24.1%) | 0.089 |
| Smoking (%) | 1239(40.9%) | 2231(38.4%) | **0.026** |
| Depressive symptoms score | 9.8±5.5 | 8.5±7.1 | **<0.001** |
| Elementary school education or above(%) | 1628(53.7%) | 2908(49.9%) | **<0.001** |
| **Laboratory results** |  |  |  |
| eGFRcr-cys (mL/min/1.73 m^2^) | 87.6±14.6 | 82.6±19.5 | **0.001** |
| Uric acid (mg/dL) | 4.4±1.2 | 4.5±1.3 | **0.001** |
| Glucose (mg/dL) | 109.2±32.4 | 110.4±38.4 | 0.595 |
| Glycated Hemoglobin (%) | 5.3±0.8 | 5.3±0.8 | 0.843 |
| Total cholesterol (mg/dL) | 192.7±37.4 | 194.2±37.5 | 0.062 |
| HDL cholesterol (mg/dL) | 51.4±15.3 | 50.9±15.3 | 0.176 |
| LDL cholesterol (mg/dL) | 117.3±34.6 | 116.0±35.2 | 0.091 |
| Triglycerides (mg/dL) | 132.1±97.5 | 133.3±105.1 | 0.584 |

Notes:

Data are shown as means ± standard deviation or numbers (percentages).

*p Values correspond two-tailed t-test for continuous variables and chi-squared test for categorical variables.

**Supplemental Table 2. The relationships of social isolation and its components with CKD onset.**

|  | Events/N (%) | Model 1 | | Model 2 | | |
| --- | --- | --- | --- | --- | --- | --- |
|  |  | OR (95% CI) | P value | OR (95% CI) | | P value |
| **Social isolation components** |  |  |  |  |  | |
| **Not married** |  |  |  |  |  | |
| No | 63/2441(2.6%) | (Reference) |  | (Reference) |  | |
| Yes | 24/590(4.1%) | 1.420(0.870-2.318) | 0.161 | 1.289(0.723-2.299) | 0.390 | |
| **Less than weekly contact with children (%)** |  |  |  |  |  | |
| No | 79/2726(2.9%) | (Reference) |  | (Reference) |  | |
| Yes | 8/305(2.6%) | 0.758(0.426-1.862) | 0.758 | 1.049(0.493-2.232) | 0.900 | |
| **Live in the rural area (%)** |  |  |  |  |  | |
| No | 10/549(1.8%) | (Reference) |  | (Reference) |  | |
| Yes | 77/2482(3.1%) | 1.523(0.964-2.405) | 0.072 | 1.692(0.798-3.585) | 0.170 | |
| **Not participate in social activities (%)** |  |  |  |  |  | |
| No | 38/1591(2.4%) | (Reference) |  | (Reference) |  | |
| Yes | 49/1440(3.4%) | 1.750(0.899-3.406) | 0.100 | 1.285(0.802-2.058) | 0.297 | |
| **Live alone (%)** |  |  |  |  |  | |
| No | 63/2447(2.6%) | (Reference) |  | (Reference) |  | |
| Yes | 24/584(4.1%) | 1.442(0.883-2.352) | 0.143 | 1.311(0.735-2.341) | 0.359 | |

Model 1 was adjusted for eGFR at baseline. Model 2 was adjusted for age, sex, body mass index, smoking status, systolic BP, diastolic BP, glucose, total cholesterol, triglycerides, HDL cholesterol, eGFR, uric acid, and the depressive symptoms score.
